# Integration of color and intensity increases time signal stability for the human circadian system when sunlight is obscured by clouds

**DOI:** 10.1038/s41598-018-33606-5

**Published:** 2018-10-12

**Authors:** T. Woelders, E. J. Wams, M. C. M. Gordijn, D. G. M. Beersma, R. A. Hut

**Affiliations:** 1 Chronobiology unit, Groningen Institute for Evolutionary Life Sciences, University of Groningen, PO box 11103, 9700CC Groningen, The Netherlands; 2 Chrono@Work, B.V., Friesestraatweg 213, 9743 AD Groningen, The Netherlands

## Abstract

The mammalian circadian system encodes both absolute levels of light intensity and color to phase-lock (entrain) its rhythm to the 24-h solar cycle. The evolutionary benefits of circadian color-coding over intensity-coding per se are yet far from understood. A detailed characterization of sunlight is crucial in understanding how and why circadian photoreception integrates color and intensity information. To this end, we continuously measured 100 days of sunlight spectra over the course of a year. Our analyses suggest that circadian color-coding may have evolved to cope with cloud-induced variation in light intensity. We proceed to show how an integration of intensity and spectral composition reduces day-to-day variability in the synchronizing signal (*Zeitgeber*). As a consequence, entrained phase angle of the circadian clock will be more stable, which will be beneficial for the organism. The presented characterization of sunlight dynamics may become important in designing lighting solutions aimed at minimizing the detrimental effects of light at night in modern societies.

## Introduction

Mammals possess a circadian oscillator, located in the suprachiasmatic nucleus (SCN) of the hypothalamus. On their own, circadian clocks rarely cycle with a period of exactly 24 hours^1^. For the circadian oscillator to serve as a reliable internal representation of the light-dark cycle, a stable phase relationship is required between the solar cycle and the circadian rhythm. This is realized via entrainment; as first described by Christiaan Huygens illustrating how two interacting oscillators assume a common period and stable phase relationship^2^. The circadian oscillator system interacts with the solar cycle via its sensitivity to light; the most potent time-cue (i.e *‘Zeitgeber’*, german for time-giver) for circadian timekeeping. Light accelerates or decelerates the cycling rate of the circadian oscillator, with direction and magnitude depending on the timing of light reception. Light in the early morning typically accelerates the clock, whereas light in the late evening slows it down^3^. This response is positively related to light intensity^4,5^. Entrainment occurs when the phase-relationship between the circadian oscillator and the solar cycle is stable, such that the daily integrated effect of light on the cycling-rate of the clock makes up for the difference that exists between the periods of the daily solar cycle (T) and the circadian oscillator (τ). The phase-angle difference between the solar cycle and the circadian oscillator satisfying this condition is known as the phase angle of entrainment. In the average human, the phase angle of entrainment would ensure a daily acceleration of 0.2 hours^6,7^.

Thus, circadian clocks have evolved to represent solar time by entraining to a signal that closely corresponds to solar time (i.e. light intensity), implying that circadian clocks have evolved to obtain maximal stability from day to day. However, considering the circadian system as an oscillator driven solely by light intensity poses a potential problem in fulfilling this purpose. First, behavior-induced noise in perceived intensity, such as moving in and out of shade, is a problem that needs to be addressed by a circadian photoreceptive system^8^. Such noise is partially accounted for by integrating light intensities over time, resulting in low-pass filtering of the *Zeitgeber* signal^9^. Another possible strategy by which such changes may be accounted for is through common-node noise rejection^10^, by making use of another signal that normalizes for such changes, such as color (unlike intensity changes by burrow visits^8^, color and intensity may change in concert when moving in and out of vegetation shade, such that the perceived change in color may be used to correct for the perceived change in intensity). Importantly, not only behavior induces *Zeitgeber* noise, but also external factors such as cloud cover impact the perceived intensity of sunlight, which will be the main focus of the work presented here. For example, during an overcast evening following clear-sky days, the delaying effect of light will be smaller than usual, which would cause an unwanted advance. It has therefore been proposed that circadian systems have evolved color-vision to obtain a more accurate indication of solar time^8,10–14^. This is plausible, as wavelength-dependent photon scattering in the atmosphere depends on solar angle, such that distinct changes in the color of the sky may be observed around dusk and dawn^15^. However, whether color indeed provides additional information on solar time over intensity *per se* remains unclear. Additionally, how color and intensity information can be integrated to maximize *Zeitgeber* stability has not been systematically described. Therefore, we collected 100 days of omnidirectional sunlight spectra over the year under varying weather conditions to provide data-driven evidence that an integration of color and intensity provides more information on solar time than intensity alone for the human circadian system.

## Methods and Materials

### Data collection

Measurements were performed on the roof of the Bernoulliborg, a 27 meters high building located on the Zernike campus of the University of Groningen, the Netherlands (latitude: 53.24°, longitude: 6.54°), providing clear measurements of the horizon. As our parameter of interest was the perceived sunlight spectrum irrespective of the direction of gaze, we decided to measure spherical (or scalar) irradiance, which is defined as the irradiance on a point where all directions are weighted equally^16^. To this end, a special designed spherical milk glass diffusor was used (4π diffusor), which was designed to project incident light from any direction onto an inserted cosine corrected diffusor mounted on a 5 m long fiber optics cable. Importantly, this sensor efficiently captures light from angles close to the horizon, which is required given our interest in color changes around dusk and dawn^15^. The fiber optics cable was used to guide incoming light onto the light-sensitive element of a SpecBos 1211 LAN UV spectroradiometer (JETI Technische Instrumente GmbH, Germany). We refer to sunlight as the global irradiance (consisting of direct, diffuse and scattered sunlight), with a slight but minimal contribution of light reflected from vegetation from the surface of the earth in the upward direction, as the spectrometer was placed on a large concrete surface. The setup, which was fully calibrated by the manufacturer, thus allowed for the measurement of irradiance from all directions simultaneously, except for the part of the sphere blocked by the diffusor part and fiber optics cable. For semi-continuous sampling, measurements were programmed to start automatically each full minute. The integration time of the device was set to automatic mode, meaning that the duration of each measurement was inversely related to the intensity of the captured light to avoid under- or overexposure of the light-receiving element. Consequently, the sampling frequency of once per minute was reduced during darkness. A total of 88960 samples were acquired over the time course of 100 days throughout the year (11, 30, 22 and 37 spring, summer, autumn and winter days respectively). The reason for this uneven distribution is that the photospectrometer was not always available, as it was shared with other members of the research group. Each sample contained one irradiance value (W/m^2^) for each wavelength within the 380–780 nm range at a resolution of 1 nm. All light intensity data were expressed in photons cm^−2^ s^−1^ and further processed in *R* (version: 3.2.3), using the *RStudio* shell (version: 1.0.136). Solar angle was calculated according to Michalsky’s algorithm^17,18^. The details of the calculations used to convert time to solar angle can be found in the *SolarAngle R* script in our publicly available fileset (see ‘open source dataset and *R* code’).

### Setup validation

Human color discrimination occurs via a well-described comparison between the activation of short-, mid- and long-wavelength cones (S-, M- and L-cones respectively) in the human retina^19^. For any stimulus, its perceived color can be numerically described, typically by calculating the two CIE 1931 *xy* chromaticity coordinates from the associated irradiance spectrum. For validation purposes, we calculated the CIE 1931 *xy* chromaticity coordinates of all samples, together with the total irradiance measured per solar angle integrated over the 380–780 nm wavelength range (see Fig. S1). These values corresponded closely to previously reported values using sunlight measurements in an urban setting^15^. As expected from these published data, light pollution was apparent in the dataset until solar elevations exceeded −6° over the horizon as measurements were performed on the edge of an urban environment (Fig. S2). Therefore, solar angles below −6° were discarded from the data, leaving a range of −6° to 60° for analysis.

### Human circadian photometrics

In the mammalian retina, light information is conveyed to the SCN via a specialized subset of retinal ganglion cells that encode irradiance via expression of the melanopsin photopigment. Both, in primates (macaques) and rodents, these intrinsically photosensitive retinal ganglion cells (ipRGCs) have additionally been shown to receive indirect synaptic input from cones, such that spectral changes modulate the ipRGC response in addition to changes in absolute intensity^12,20,21^. In the macaque retina, S-cones exert an inhibiting effect on the ipRGC response and M- and L-cones together fulfill an excitatory role^12^. For human circadian photoreception, evidence regarding the role of cones in circadian entrainment is not conclusive, posing a potential problem for calculating the circadian equivalent of visual chromaticity coordinates. However, given the remarkable similarity of color-coding in human and macaque retinae^22^, together with recent findings that the human pupillary control system encodes color information in a manner very similar to what was reported for macaques^23–25^, we decided to express color on the +S/(L + M) coordinate of the MacLeod-Boynton chromaticity diagram, such that color is expressed on a blue-yellow axis^26^. Cone excitations were calculated using the CIE 170-1:2006 10° cone fundamentals^27^, which account for lens, macular pigment and effective photo pigment optical densities. For the estimation of photons absorbed by the melanopsin photo pigment (photons cm^−2^ s^−1^), the spectra were manually corrected to account for the optical density of the human lens for the average 32-yr-old observer^28^. We then calculated the estimated absorbed photons by melanopsin using a Govardovski^29^ nomogram with λ_max_ set to 480 nm at a photo pigment optical density of 0.015.

### Data preparation for solar angle estimations

The integration time of the spectrometer increases at lower light levels (lower solar angles) to ensure reliable spectral intensity measurements. This resulted in non-uniform data distribution over each solar angle, which may induce modelling artifacts due to an underrepresentation of samples at low solar angles. Therefore, a resampling procedure was performed to obtain a uniform distribution of solar angles over the complete dataset. The dataset was first divided into 1-degree solar angle bins. The target sample size of each bin was then set to the size of the largest bin (2382 spectral measurements per solar angle bin) and resampling was performed until each bin reached the target sample size, resulting in a uniform sampling distribution over each solar angle. Thus, the uniform dataset contained 159594 samples, divided over 67 solar angle bins, with 2382 samples per bin.

### Predictive modelling of solar angle by color and intensity

To model the nonlinear relationship between solar angle, color and intensity, a *k*-nearest neighbors (KNN) machine learning approach was employed. KNN models are non-parametric; no *a priori* assumptions are made on the relationship between the explanatory and dependent variables. This minimizes the possibility of incorrectly modelling the underlying data distribution, and is therefore well-suited to reveal nonlinear patterns in the data. Before constructing a KNN-model, the dataset is usually partitioned into a training set and a test set. The predicted solar angle for each test sample is the average of its *k* nearest neighbors from the training set (in our case the closest Euclidean distances in the intensity vs. color plane). The model was first trained on the training set (10% of the total data) to determine the optimal value for *k*, using repeated (3 times) *K*-fold (10 times) cross-validation. In this process, the training data is partitioned into *K* (*K* = 10, not to be confused with *k*) equally sized randomly chosen subsets for each value of *k* (1 to 100 in steps of 1), where *K*-1 subsets are combined and used as training data and the remaining subset is used as validation data. This step is then performed *K* times, each time using a different part of the data (10%) as the validation set, resulting in 10 models for each value of *k*. This procedure was additionally performed 3 times to maximize reliability of the fitting procedure, each time randomly assigning different samples to the 10 subsets, such that for each value of *k*, 3 × 10 models were fit. Performance of the *k* models was evaluated from the root mean square error (RMSE) between predicted and observed solar angles. The average RMSE was calculated for each value of *k*. For the model incorporating intensity and color (combined model), the parameter value *k* of 35 described predictable patterns in the dataset most accurately (i.e. lowest average RMSE). This model was then used to assign an estimated solar angle to each sample in the uniform dataset based on color and intensity. Two additional models were constructed to assess the predictability of solar angle from intensity (intensity-only model) or color (color-only model) alone (optimal *k* = 100). In the case of the combined model, the procedure is effectively a smoothing process, and reveals patterns in the color vs. intensity plane that are mostly associated with particular solar angles. KNN models were trained using the *caret* library (version: 6.0.80) in *R*. As KNN models are typically fitted to normalized data, color and log melanopic photons were normalized by z-transformation before modelling, yielding an average of 0 and a standard deviation of 1 for these metrics.

### Weather conditions

Cloud cover information for the measurement days was obtained from the Dutch national meteorological institute (KNMI). A KNMI weather station is located at Groningen Airport Eelde, the Netherlands (latitude: 53.13°, longitude: 6.59°), at a distance of 14 km from our spectral measurement location, which keeps records on the average cloud cover amount per day. This station is equipped with a cloud-sensor that works according to the light detection and ranging (LIDAR) principle. Once every 15 seconds, the sensor sends an infrared signal vertically into the atmosphere and determines the presence of a cloud depending on the recurrent signal. The total cloud cover amount over a 24-hour day is then calculated from the percentage of cloud-cover detections over a day. Cloud cover (i.e. KNMI index) is expressed on a linear 0–8 scale (i.e. ‘octas’), where a value of 8 means that at every sample over the 24-hour day the sky was not detectable, whereas a value of 0 indicates that at none of the samples a cloud was detected.

### Statistical procedures

Linear regression models were constructed in *R*, using the native *lm* function, where a significant contribution of individual terms was determined using the *drop1* function. A critical p-value of 0.05 was maintained for these analyses. Two-dimensional density distributions were calculated for data visualization using the Two-Dimensional Kernel Density Estimation function (*kde2d)* from the *MASS* library (version: 7.3–49) in *R*. These 2D-density distributions were calculated from the uniform dataset. For data visualization of the combined model, a smoothed relationship between color and intenisty was obtained for five representative solar angles by local polynomial regression fitting (native *R loess* function)^30^.

### Construction of a solar angle-dependent dose-response curve

For construction of the solar angle-dependent dose-response presented in Fig. S3, we made use of the human dose-response curve published by Zeitzer and colleagues (2000)^4^. We first shifted the sensitivity of the original dose-response curve to a higher intensity, to approximate the sensitivity of a light-adapted human (Fig. S3a). The motivation to do this, is that in that study, individuals were exposed to a light pulse after multiple days in dim light conditions which likely sensitized the circadian system by about 0.5 log units^31^. Then, we considered the relationship between intensities on the log melanopic photon and lux scales in our dataset, where the highest measured intensities corresponded to intensities of ~120 Klux. It is not likely that human eyes are ever exposed to such intensities (which mainly originate from direct sunlight), and a more plausible approximation of corneal light intensity for a human is the intensity of diffuse sunlight, which is about 1 log unit lower than direct sunlight^32^ (Fig. S3b). Based on the linear relationship between log melanopic photons and corneal lux (1 log unit subtracted from the global lux values), we converted the x-axis of the dose-response curve to log-melanopic photons (Fig. S3c). Finally, based on the modelled relationship between log melanopic photons and solar angle of the intensity-only model, we converted the intensity units on the x-axis to solar angle (Fig. S1d). This way, we were able to estimate a normalized response of the human circadian system to the predicted solar angles of the color-only, intensity-only and combined models separately.

### Open source dataset and *R* code

The calibrated data and the *R* code used to construct the figures and to perform all analyses are available at, 10.6084/m9.figshare.7033460.

## Results

### Solar angle-dependent effects of cloud cover on the sunlight spectrum

**Figure 1:**
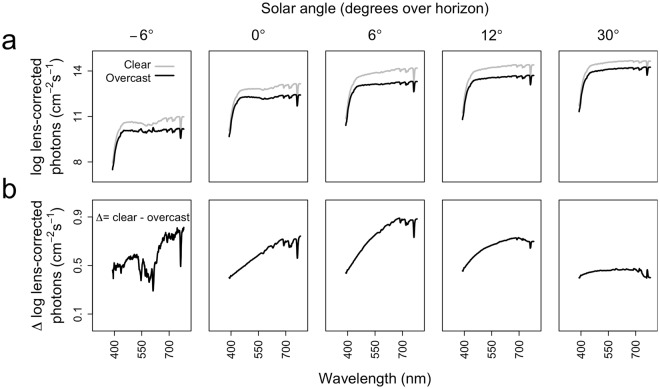
Progression of sunlight power spectra. (**a**) *P*rogression of sunlight power spectra for 5 representative solar angles, for 11 clear (KNMI index < 3) and 43 overcast (KNMI index > 6) days. **(b)** Difference between power spectra at clear and overcast skies for 5 representative solar angles.

Retinal irradiance (i.e. lens-corrected photons) increases with increasing solar angles (Fig. 1a), but the decrease of lens-corrected photons by cloud cover, depends on wavelength and solar angle (Fig. 1b). Typically, incident photons of shorter wavelengths are less affected by cloud cover than photons of longer wavelengths, especially during lower solar angles (−6° to 12°).

### Effects of cloud cover on encoded intensity and color

**Figure 2:**
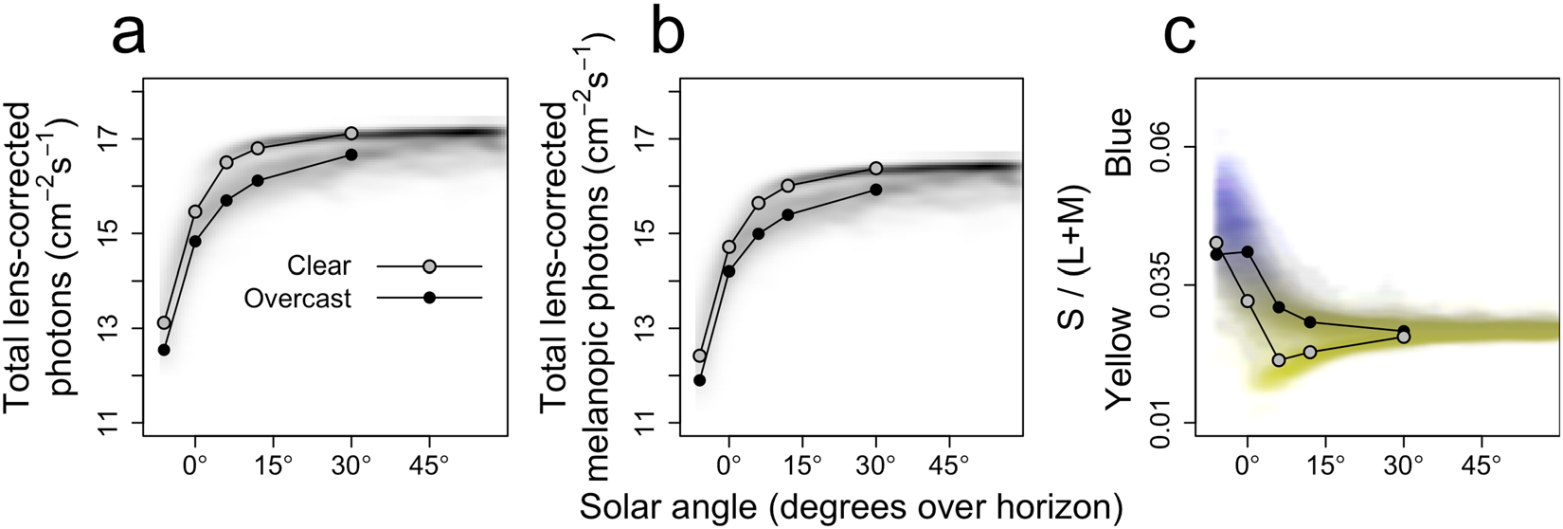
Density profiles of intensity and color against solar angle. 2D density profiles (see methods) of the total dataset for solar angle against **(a)** total lens-corrected photons, **(b)** total lens-corrected melanopic photons and **(c)** color. Higher transparency indicates a lower density of samples. Samples with colors higher or lower than average are displayed in blue or yellow respectively, with the purest hues at the extremes. Overlays are drawn for the average clear (n = 11; KNMI index < 3) and overcast (n = 43; KNMI index > 6) days.

**Figure 3:**
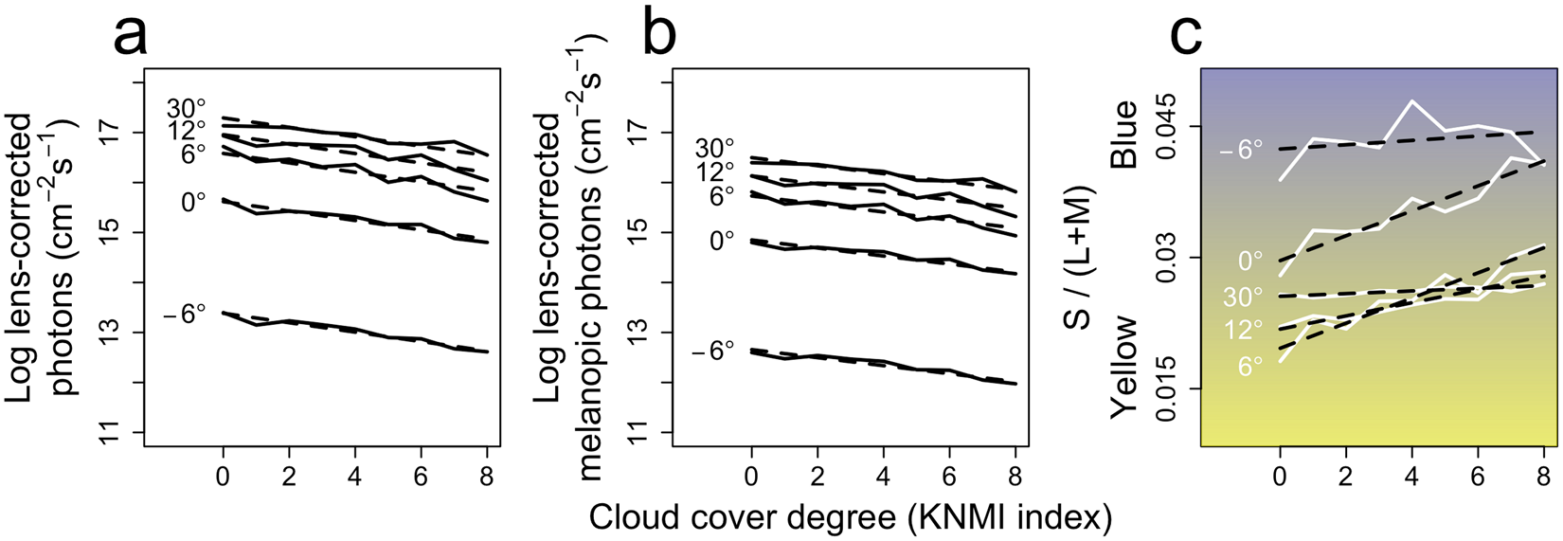
Effects of cloud cover on intensity and color. Effect cloud cover on **(a)** total lens-corrected photons, **(b)** total lens-corrected melanopic photons and **(c)** color. Dashed lines represent linear regression model fits.

To quantify how these dynamics in sunlight spectra are processed by the human retina, the effect of solar angle and weather conditions on circadian light intensity (i.e. log melanopic photons) and color were determined. The log-transformed integration (380–780) of human lens-corrected photons was included as a reference. As expected, the total number of lens-corrected (melanopic) photons increased with increasing solar angles, and at each solar angle, cloud cover decreased the amount of (melanopic) photons (Fig. 2a,b). On average, color decreases with increasing solar angles, indicating a transition from blue to yellow (Fig. 2c). Clear skies are typically relatively yellow compared to overcast skies, especially within the 0–30° solar angle range. We then determined the effects of various amounts of cloud cover on the lens-corrected (melanopic) photons (Fig. 3a,b) and color (Fig. 3c). Linear regression analyses revealed a significant main effect of cloud cover amount on lens-corrected photons (F(5,39) = 1884, p < 0.001) and lens-corrected melanopic photons (F(5,39) = 2417, p < 0.001). Each additional increase of cloud cover (on KNMI scale 0 (clear sky) to 8 (sky not visible)), decreased the number of lens-corrected and melanopic photons by 0.09 ± 0.04 and 0.08 ± 0.03 (±SEM) log photons respectively. An interaction effect was observed when explaining color by both the amount of cloud cover and solar angle (F_9,35_ = 121, p < 0.001). Typically, more cloud cover was related to bluer skies, an effect that was especially pronounced at solar angles between 0° and 12° (Fig. 3c). These analyses reveal that there is a solar angle-dependent effect of cloud cover on sunlight color, where clouds cause a blue shift of the sunlight spectrum especially at lower solar angles. This could potentially provide the circadian system with the information necessary to obtain a solar angle-dependent *Zeitgeber* signal regardless of cloud conditions.

### Solar angle predictions

**Figure 4:**
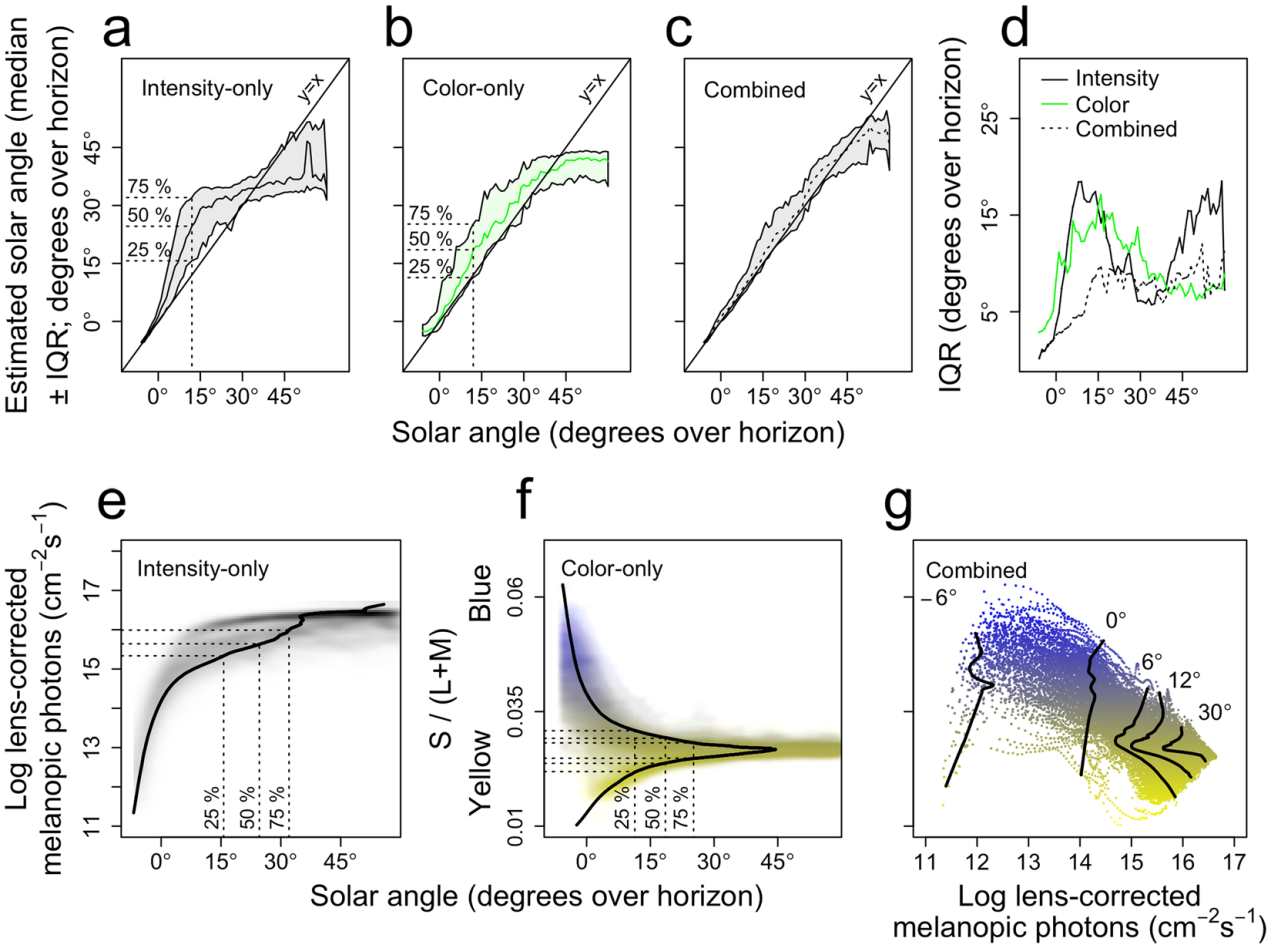
Solar angle estimations by intensity-only, color-only and combined models. Solar angle estimations ± inter quartile range for the **(a)** intensity-only, **(b)** color-only and **(c)** combined models. The dashed projections illustrate the 25-, 50- and 75-percentiles of the estimated solar angles at an example actual solar angle of 12°. **(d)** inter quartile range of estimated solar angles per actual solar angle for all models. **(e)** and **(f)** estimated solar angle per intensity and color (solid black lines) overlayed on the 2D density profiles (see methods). The vertical dashed projections correspond to 25-, 50- and 75-percentiles of the estimated solar angles at an actual solar angle of 12° [from **(a)** and **(b)**]. The horizontal dashed projections illustrate the translation from these estimated solar angles to intensity and color values. **(g)** Smoothed color-intensity relationships (*loess* fit; see methods) for five representative estimated solar angles overlaid on the color versus intensity plane.

Figure 4a shows the median ± inter-quartile range (IQR) of the solar angle estimations per solar angle for the intensity-only model. Figure 4e shows the estimated solar angle per intensity for the intensity-only model, which is for each intensity the solar angle at which that intensity occurred most frequently. For example, intensities of 15.3, 15.6 and 16 log melanopic photons most frequently occur at solar angles of 15.7°, 24.6° and 32° respectively (Fig. 4e). It is important to note that these intensities are not necessarily the intensities that were most frequently observed at those solar angles. For example, at a solar angle of 12°, 50% of the solar angle estimations of the intensity-only model are between 15.7° and 32° around a median angle of 24.6° (Fig. 4a, horizontal projections). This positive bias is not a modelling artefact, but it originates from the fact that 50% of the intensities observed at a solar angle of 12° are between 15.3 and 16 log melanopic photons (Fig. 4e, horizontal projections). These intensities most frequently occur at higher solar angles (Fig. 4e, vertical projections), as predicted by the intensity-only model, resulting in the overestimation observed at a solar angle of 12° (Fig. 4a). These data thus illustrate how using intensity information only to estimate solar angle leads to underestimations or overestimations depending on the distribution of the intensities at different solar angles. Similarly, 50% of the solar angle estimations of the color-only model are between 11.3° and 25.2° around a median of 18.5° (Fig. 4b). This occurs because 50% of the colors are between 0.028 and 0.031 or 0.022 and 0.025 (bimodal distribution; Fig. 4f, horizontal projections). These colors typically occur at higher solar angles (Fig. 4f, vertical projections), as predicted by the color-only model, resulting in the overestimation observed at a solar angle of 12° (Fig. 4b). The combined model shows relatively little bias, as the relation between estimated and actual solar angle deviates little from *y* = *x* (Fig. 4c). The low-bias and low-variability estimations of the combined model indicate that, unlike color or intensity alone, specific combinations only occur within narrow ranges of solar angles. Therefore, the combined model predictions reveal the patterns in the color versus intensity plane that are typically associated with different solar angles (Fig. 4g), irrespective of overcast conditions. For example, at a solar angle of 12°, yellow, intermediate and blue colors are associated with high, low and intermediate intensities, respectively (Fig. 4g). These different clusters reflect samples obtained under clear skies, completely overcast skies and partially overcast skies where direct sunlight is blocked. From a circadian perspective, the variability is the most biologically relevant measure of performance; a biased estimation with low variability will provide the same day-to-day stability in a derived *Zeitgeber* signal as an unbiased estimation with the same variability. The variability in estimated solar angle is therefore presented for all solar angles and for all models in Fig. 4d. Especially between 0° and 20°, the combined model provides less variable solar angle estimations than the intensity-only and color-only models. At higher solar angles above 40°, the combined model outperforms the intensity-only model but is slightly more variable than the color-only model. This is because the intermediate colors that are observed at these higher solar angles all result in similar (although biased) solar angle estimations (Fig. 4b). Similarly, at a solar angle of 30°, the intensity-only model slightly outperforms the combined model because most intensities at that solar angle are clustered around 16.3 log melanopic photons, resulting in biased but low-variability solar angle estimations at a median of 35°. Overall, the combined model performs equally well or better, especially at solar angles between 0° and 20°. Of note is the small number of yellow low-intensity points in Fig. 4g. These likely reflect light pollution, and more precisely the fact that with decreasing solar angles beyond −6°, the color of the sky becomes again more yellow, which occurs earlier in city than rural areas^15^. These points should therefore be interpreted with caution.

### Increasing *Zeitgeber* stability by estimating solar time

**Figure 5:**
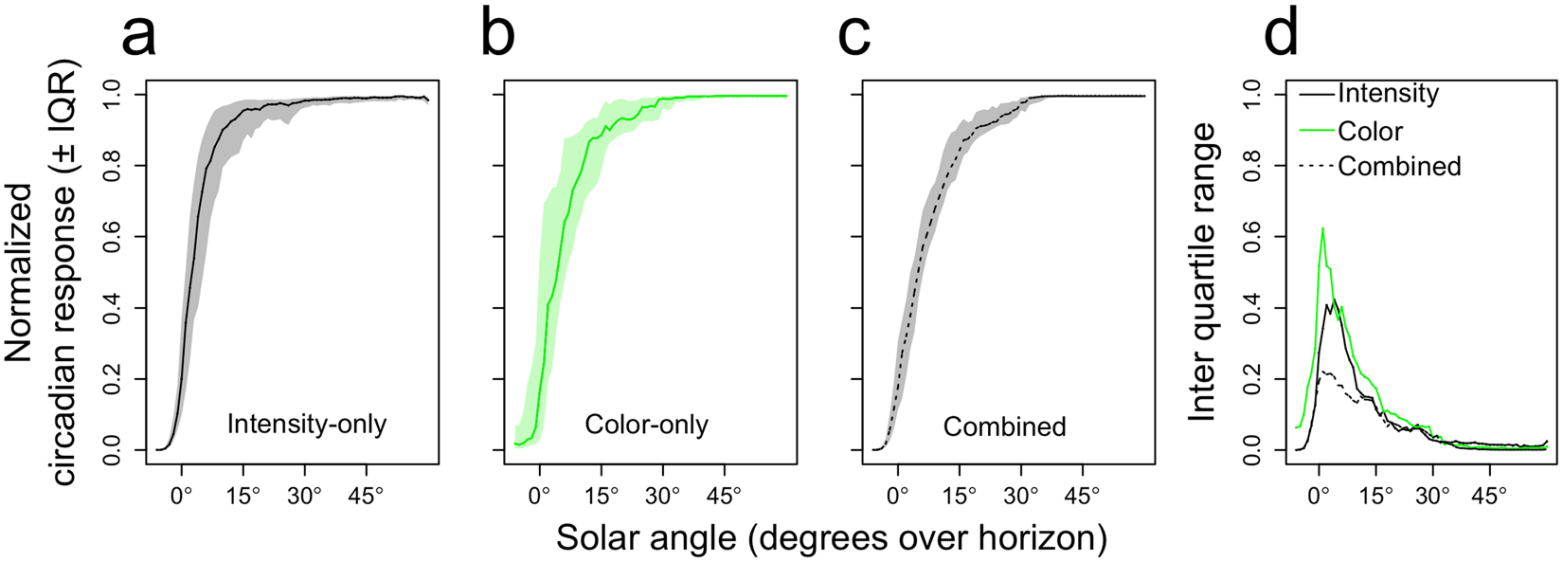
Accurate solar angle estimations minimize day-to-day variability in predicted circadian response. The normalized predicted circadian response ± inter quartile range for the **(a)** intensity-only, **(b)** color-only, and **(c)** combined models. **(d)** Inter quartile range of normalized circadian response per actual solar angle, for all models.

We next turned to the question as to how such estimations of solar angle may be translated into a *Zeitgeber* signal that is more stable and provides more information on solar time than a signal depending solely on light intensity or color. To this end, we estimated the distribution of estimated solar angles per 1-degree solar angle bin, for the intensity-only, color-only and combined models separately. In addition, we wished to account for the fact that the human circadian system saturates at higher light intensities (and consequently higher estimated solar angles). We therefore first constructed (from a published dose-response curve (DRC) for the human circadian system^4^) a DRC of the human circadian system with estimated solar angle as the independent variable instead of light intensity, using the relationship between light intensity and estimated solar angle based on the intensity-only model. Then, we projected the solar angle estimations of the intensity-only, color-only and combined models onto this solar angle-dependent DRC to obtain for each sample, per model, an estimated circadian response (see methods and Fig. S3a–d for a detailed description of the procedures involved). As this solar angle-dependent DRC saturates at higher estimated solar angles, the circadian response is less susceptible to noise at saturating solar angles (Fig. 5a–d). This analysis demonstrates that, although the combined model outperforms the intensity-only model at the highest solar angles (Fig. 4d), this need not be beneficial for a system that saturates at higher solar angles (Fig. 5d).

## Discussion

Although the theoretical notion that color alone may be a more precise *Zeitgeber* than light intensity around dusk and dawn has been around for some time^8,11–14^, this does not seem to be in line with our findings that the color of the sky at lower solar angles is very much influenced by cloud-cover, arguably more so than light intensity. The fact that color was found to be highly variable at solar angles between 0° and 30° (Figs 2c and 4f) is explained by the interaction of sunlight with the atmosphere and clouds^33,34^. Due to Rayleigh scattering of mainly short-wavelength photons in the atmosphere, these photons reach the sensor before sunset in the form of indirect (i.e. diffuse) sunlight, causing a predominantly blue spectrum. The relatively weak effect of Chappuis filtering by ozone (400–650 nm with a maximium at ~575–603 nm)^35^ is also visible at these lowest solar angles (Fig. 1a, solar angles −6° and 0°). The relatively high-intensity direct sunlight beam^32^ causes a long-wavelength shift in the spectrum directly after sunrise (Figs 1b and 2c). As the solar angle increases further, the short-wavelength component in the direct sunlight beam increases as light travels a shorter distance through the atmosphere, resulting in less filtering of short-wavelength photons out of the direct sunlight beam, shifting the spectrum towards blue again (Figs 1b and 2c). On overcast days, only a blue-yellow progression is observed, which is likely due to the additional interaction of photons with cloud droplets by Mie scattering^33,34^. Just after sunset, the direct sunlight beam hits the top of the cloud at a small angle, such that direct sunlight travels a relatively large distance through the clouds, decreasing the contribution of the long-wavelength shifted direct sunlight component. Much of the diffuse short-wavelength shifted sunlight reaches the clouds perpendicularly, allowing for a relatively efficient transmission of mainly short-wavelength photons. At higher solar angles when the incident angle of direct sunlight increases, this effect finally becomes negligible. In addition, the shape of the spectrum is determined by the location of clouds in the sky hemisphere. Partially overcast conditions when mainly diffuse sunlight is visible result in the bluest spectra, whereas the yellowest spectra are observed when only the direct sunlight beam is visible (Fig. 4g). Our observation that the color is so variable at lower solar angles is thus due to direct sunlight reaching the sensor more efficiently (relative to diffuse sunlight) during lower solar angles on clear-sky days than on overcast days. Importantly, this effect can therefore only be observed when a spherical captor is used (that efficiently captures direct sunlight at low solar angles). Indeed, when solar spectra are sampled with horizontally oriented sensors that are mainly sensitive to perpendicular incident photons^13,15^, the change in color over the course of the day is very similar to the change we report for overcast days. Therefore, such set-ups underestimate the yellowness of the solar spectrum at low solar angles on clear-sky days, and as a consequence also the variability in color that can occur at those solar angles according to the physics described above.

Thus far, it has been difficult to formulate clear hypotheses regarding the evolutionary benefits of circadian color-coding, as a detailed evaluation of sunlight from a circadian perspective was lacking. Our analyses show that predictable weather- and solar angle-induced effects on both the color and intensity of sunlight (Fig. 4g) are prime candidates to be used by circadian clocks to acquire maximal information on solar time. We therefore conclude that an integration of color and intensity provides the most accurate *Zeitgeber* that the human eye can extract from sunlight (Figs 4d and 5d). Such circadian color coding may be beneficial by allowing circadian systems to differentiate between solar angle- and weather-induced effects on the intensity of sunlight. Light intensity decrease under an overcast sky in the evening, would reduce its circadian phase delaying effect at that time of day, thus generating an unwanted advance in circadian phase angle of entrainment. When the *Zeitgeber* strength in the evening however corresponds to the encoded solar angle based on both color and intensity, the *Zeitgeber* may be buffered against such weather-induced noise, preventing such an advance, and therefore maximizing day-to-day stability in phase of entrainment. We demonstrate that the necessary information to adopt such strategies is present in sunlight spectra, as although both color and intensity are simultaneously affected by overcast, the resulting combination of color and intensity may still be largely unique for each given solar angle (Fig. 4g). A biological mechanism that would be able to account for effects of overcast on intensity using color, would have to modulate its circadian response at a certain intensity based on the associated color of the sky. For example, at an intensity of 15 log melanopic photons, blue and yellow colors are associated with lower solar angles (i.e. <6°) than intermediate colors (i.e. ~12°; Fig. 4g). A mechanism that alters the circadian response based on color would thus increase its response to intermediate colors at an intensity of 15 log melanopic photons, but not to blue or yellow colors.

Although we used the human photoreceptive system as a model to demonstrate the benefit of circadian color-coding at lower solar angles, other species may also benefit from circadian color-coding. For example, circadian color-coding may be even more crucial for a strictly diurnal species that does not witness dusk or dawn transitions, such as the European ground squirrel. Canonical ‘light intensity only’ circadian models cannot explain entrainment in this species^36,37^, in which all major light intensity changes are induced by its own behavior and not by dusk and dawn transitions: interestingly, this animal leaves and retreats into its burrow at a solar elevation of approximately 15° ^36,37^. A second advantage of circadian color-coding (in addition to reducing day-to-day variability in the *Zeitgeber* signal) that may be most important for such a strictly diurnal species, is that the circadian response based on both color and intensity is already expected to reliably decrease at solar angles <30° (Fig. 5c). Indeed, at least for the human circadian system, this decrease is expected to occur at much lower solar angles when only intensity is considered (Fig. 5a), which may also be true for the circadian system of the European ground squirrel. Therefore, a possible strategy for this species to entrain would be to encode solar angles below 15° as ‘solar darkness’, such that slight deviations in phase of entrainment alter the *Zeitgeber* signal that is perceived the following day, without the need for witnessing sunrise and sunset. Although this hypothesis remains to be tested, the all-cone retina of ground squirrels is indeed very well-suited for cone-driven color coding in the ipRCG pathway projecting to their SCN^38^. A limitation of our study is that solar angles <−6° were not analyzed due to light pollution (Fig. S2). However, our analyses suggest that at the lowest solar angles, color information does not benefit the human circadian system. Of course, it is still possible that this is not the case for solar angles <−6°, and rural measurements are required to test this possibility. Such measurements (while extending measurement spectra to also capture the UV range) may prove to be important for a characterization of sunlight from the perspective of nocturnal animals with UV-transmitting lenses and UV-sensitive cones. The spectral sensitivity of the mouse UV-cone (peak sensitivity at 360 nm) is well-suited to capture the earliest (or latest) diffuse sunlight that is minimally affected by atmospheric absorption by the Hartley, Huggins (200–370 nm)^39^ and Chappuis (400–650 nm)^35^ bands. Therefore, the time of day when color information is most useful for the circadian system is likely related to the sensitivity properties of the photoreceptive system of a species. In addition, inter-species variability in the dynamic range of the circadian system may further affect the range of solar angles within which the *Zeitgeber* strength can still be modulated by color information (e.g. a circadian system with a saturating response at the lowest intensities cannot use color information to estimate higher solar angles).

Evolution has likely gone a great way in minimizing the effects of noise in the *Zeitgeber* signal driving our circadian clocks in a multitude of ways. First, IpRGC cells projecting to the SCN maintain steady levels of firing until several minutes after lights-off^21^, presumably such that short instances of behavior-induced darkness (e.g. burrowing) are still accompanied by a *Zeitgeber* signal that corresponds to the solar light phase. A second strategy to reduce the impact of *Zeitgeber* noise on entrainment is the previously proposed parametric entrainment mechanism for diurnal animals, where conceptually animals entrain to an average 24-hour signal integrated over multiple days (effectively increasing the Zeitgeber signal-to-noise ratio by low pass filtering)^40^. Entraining to an averaged *Zeitgeber* signal over multiple days may be beneficial for a diurnal burrower that is typically exposed to a noisy *Zeitgeber* signal on a daily basis^37^. We suggest that this list of noise-filtering properties by circadian systems might be extended by considering circadian color- and intensity-coding as a mechanism that ensures a *Zeitgeber* signal that corresponds to the actual solar angle, which is therefore maximally stable from day to day regardless of weather conditions. Indeed, both forms of entrainment (parametric and non-parametric) in diurnal mammals will gain stability when the *Zeitgeber* signal contains as much information as possible on solar time on a daily basis. Therefore, the more erratic the light intensity profile is compared to solar angle-induced changes in light intensity, the more beneficial it may become to integrate parametric entrainment and circadian color-coding. Finally, as was discussed in the introduction, an additional hypothesis is that circadian color-coding may allow for common-mode noise rejection mechanisms^10^, such that the circadian system is buffered against behavior-induced changes in perceived intensity that are accompanied by changes in color (for example an animal moving in and out of vegetation shade).

Integration of color and intensity need not be functional for circadian entrainment only, but may in addition be used by functions displaying circannual rhythmicity. Indeed, in addition to circadian timekeeping, accurate estimation of solar time may be crucial for photoperiodic time measurement (i.e. to obtain an accurate indication of day length), which is of major importance in the timing of reproductive behavior for many species^41^. Photoperiodic time measurement in mammals relies on the same photoreceptor pathway as circadian photoreception^42,43^, which seasonal breeders employ to keep track of day length over the course of the year, allowing for breeding to occur at a time of year of abundant food-availability. Encoding sunrise and sunset with maximal accuracy may provide a means of timing reproductive behavior as accurately as possible to maximize offspring survival. According to our data, it is unlikely that animals extract time of year information directly from the sunlight spectrum, as it varies little over seasons (Fig. S4).

Recent research shows that indeed the effect of color may contribute substantially to entrainment of the mammalian (mouse) SCN^13^. Phase of entrainment in mice is altered when housed under an artificial light-dark cycle that incorporates both color and intensity changes during simulated dusk and dawn transitions, as compared to intensity modulations alone. One interpretation of these findings, which is in agreement with our postulated hypothesis, is that the strength of the *Zeitgeber* signal is modulated depending on both its color and intensity. Such studies raise the question as to whether also human phase of entrainment can be altered when indoor lighting schedules incorporate the intensity and color dynamics that are present in naturally occurring sunlight. We and others have found that human phase of entrainment is advanced with more sunlight exposure during the day^44,45^. Mimicking natural sunlight and its dynamics in indoor environments (while keeping melanopic illuminance levels as low as possible during solar darkness) may thus be beneficial in countering the delayed phase of entrainment that is so typical of humans living in modern societies^46,47^. As we expect that phase of entrainment in humans will be similar between overcast and sunny days, mimicking overcast days during day time may provide the most cost- and energy-efficient means of constructing biologically relevant indoor lighting conditions.

Given that many different species, displaying different types of behavior, may all benefit from circadian color coding (to be used for both circadian and circannual timekeeping), this topic may prove to be important to fully understand the mechanisms underlying natural circadian entrainment, especially in humans and other diurnal mammals.

## Electronic supplementary material

Supplementary Information
