# Supplementary material for: Integration of color and intensity increases time signal stability for the human circadian system when sunlight is obscured by clouds
Source: Sci Rep. 2018 Oct 12;8:15214. doi: 10.1038/s41598-018-33606-5 (PMC6185968; doi:10.1038/s41598-018-33606-5)
Supplement: Supplementary file 1 — Supplementary Information [file 41598_2018_33606_MOESM1_ESM.pdf]

Integration of color and intensity increases time signal stability for the human circadian system when sunlight is obscured by clouds - Supplementary information

Woelders, T., Wams, E.J., Gordijn, M.C.M., Beersma, D.G.M. & Hut, R.A.

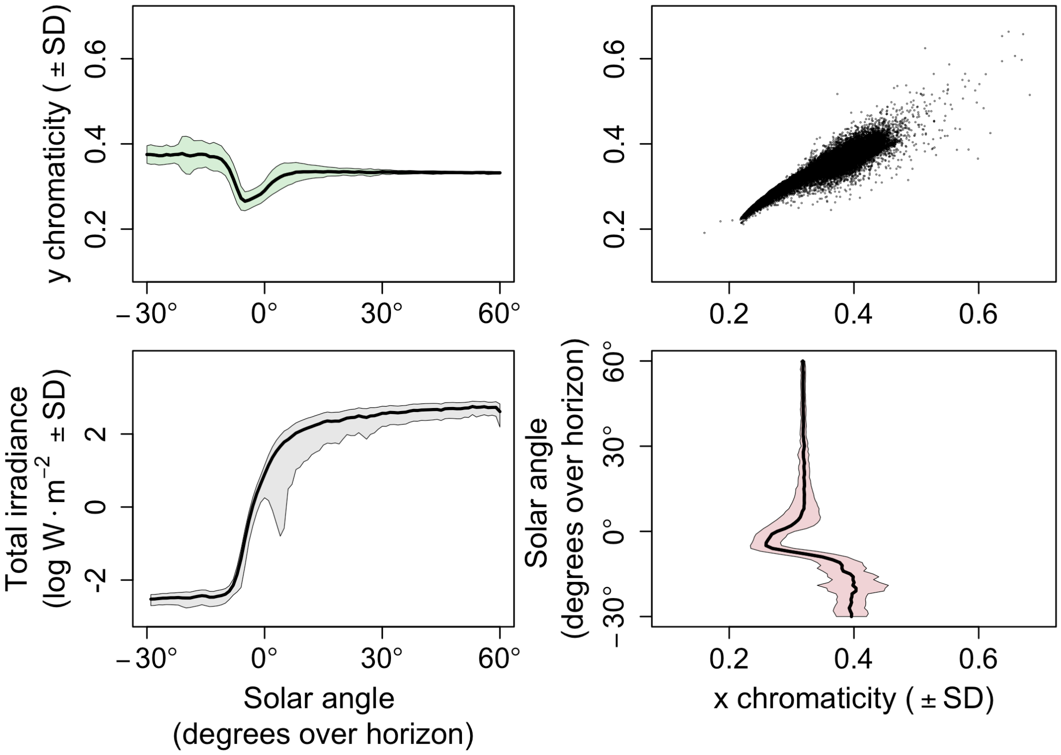

Figure S1. Progression of CIE chromaticity indices and irradiance with increasing solar angles.

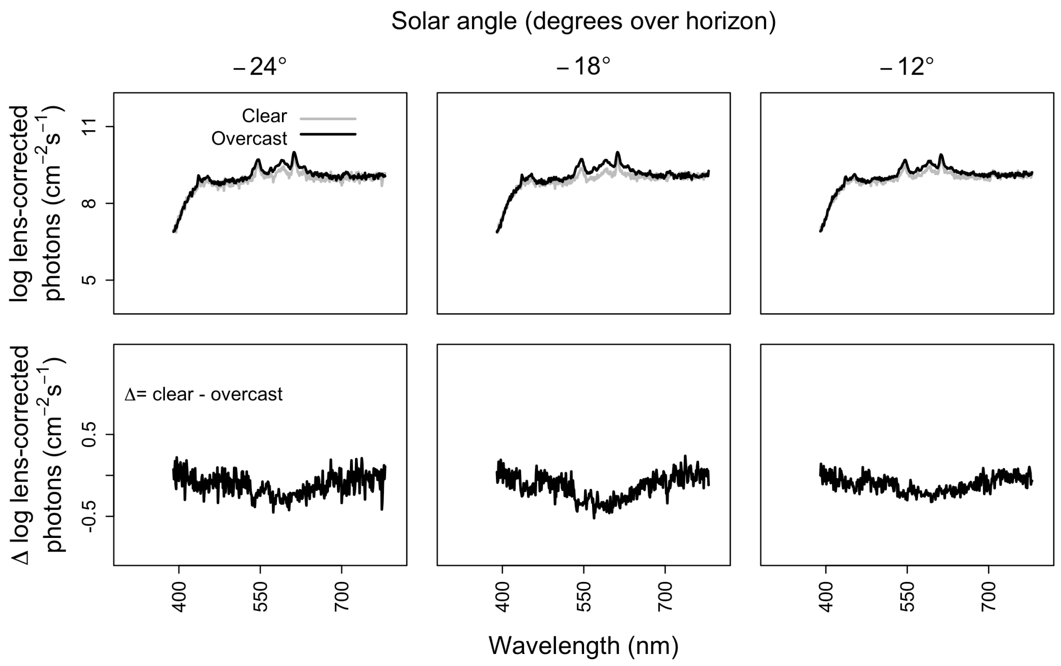

Figure S2. Sunlight spectra at lowest irradiances indicating light pollution before civil twilight. Irradiance is higher on overcast days than on clear days, indicating backscattering of city light towards the sensor.

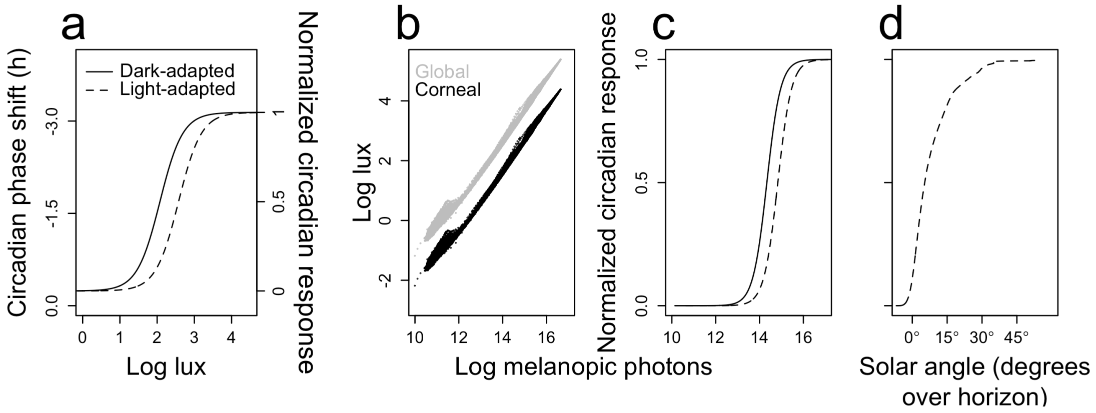

Figure S3. Construction of solar-angle dependent dose-response curve.

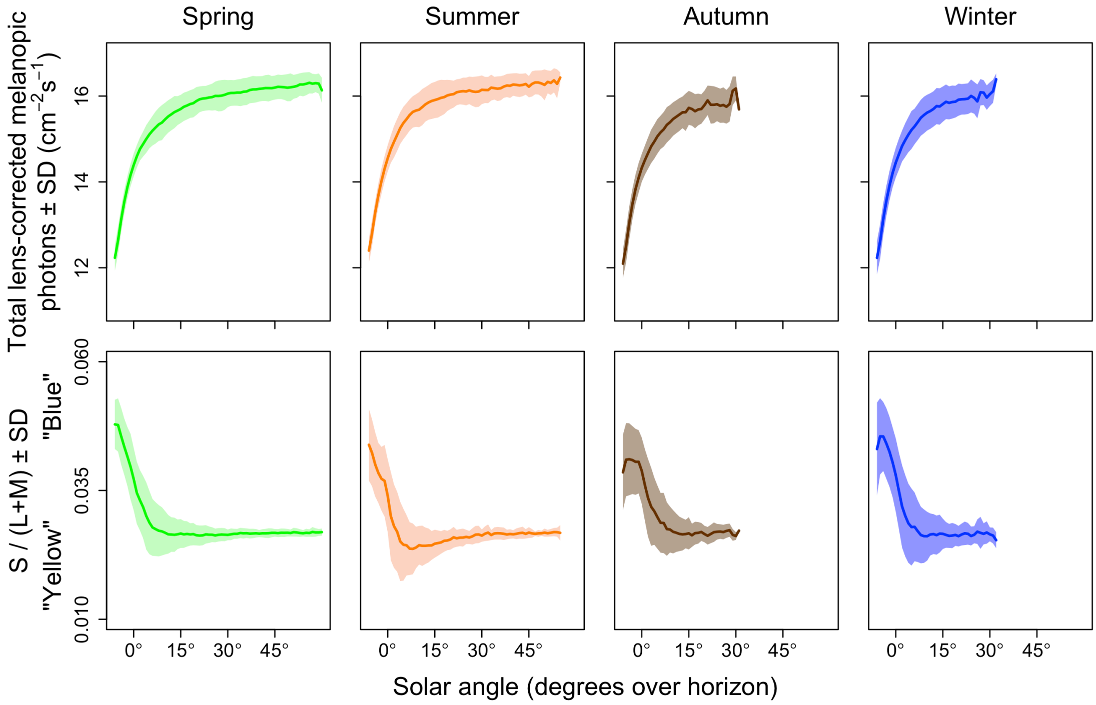

Figure S4. Irradiance and color per solar angle per season.
